# Mediating effect of BMI on the relation of dietary patterns and glycemic control inT2DM patients: results from China community-based cross-sectional study

**DOI:** 10.1186/s12889-022-14856-5

**Published:** 2023-03-10

**Authors:** Saiyi Wang, Jinbo Wen, Dandan Miao, Zhongming Sun, Dianjiang Li, Enchun Pan

**Affiliations:** 1 Huai ’an Center for Disease Control and Prevention, Huaian, 223001 China; 2 School of public health, Xuzhou Medical University, Xuzhou, 221004 China; 3 Department of Social Medicine and Health Education, School of Public Health, Nanjing Medical University, Nanjing, 211166 China

**Keywords:** Fasting Plasma Glucose, Glycosylated hemoglobin, T2DM, Dietary pattern, Mediating effect

## Abstract

**Objective:**

To analyze the effects of different dietary types on in type 2 diabetes mellitus (T2DM) and determine the mediating effects of Body Mass Index (BMI) on dietary type with Fasting Plasma Glucose (FPG), Glycosylated Hemoglobin (HbA1c) on the associations in T2DM.

**Methods:**

Data of community-based cross-sectional study with 9602 participants including 3623 men and 5979 women were collected from the project ‘Comprehensive Research in prevention and Control of Diabetes mellitus (CRPCD)’ conducted by Jiangsu Center for Disease Control and Prevention in 2018. The dietary data were collected from a food frequency qualitative questionnaire (FFQ) and dietary patterns were derived through Latent Class Analysis (LCA). Then, Logistics regression analyses were used to evaluate the associations of FPG, HbA1c with different dietary patterns. The BMI (BMI = height/weight^2^) was used as a moderator to estimate the mediating effect. Mediation analysis was performed using hypothetical variables, the mediation variables, to identify and explain the observed mechanism of association between the independent and dependent variables while the moderation effect was tested with multiple regression analysis with interaction terms.

**Results:**

After completing Latent Class Analysis (LCA), the dietary patterns were divided into three categories: TypeI, TypeII, TypeIII. After adjusting for confounding factors such as gender, age, education level, marital status, family income, smoking, drinking, disease course, HDL-C, LDL-C, TC, TG, oral hypoglycemic drugs, insulin therapy, Hypertension, Coronary heart disease, Stroke, Type III were all significantly associated with HbA1c compared to those with Type I (*P* < 0.05), and the research showed the patients with Type III had High glycemic control rate. Taking type I as the reference level, the 95% Bootstrap confidence intervals of the relative mediating effect of TypeIII on FPG were (-0.039, -0.005), except 0, indicating that the relative mediating effect was significant (α_III_ = 0.346*, β_IIIFPG_ = -0.060*). The mediating effect analysis was performed to demonstrate that BMI was used as a moderator to estimate the moderation effect.

**Conclusions:**

Our findings demonstrate that consuming Type III dietary patterns associates with good glycemic control in T2DM and the BMI associations would be playing a two-way effect between diet and FPG in Chinese population with T2DM, indicated that Type III could not only directly affect FPG, but also affect FPG through the mediating effect of BMI.

## Introduction

Diabetes has become a serious public health problem worldwide, the International Diabetes Federation (IDF) reported about 463 million with diabetesandaccounting for 9.3%in 2019,and the prevalence would from to 578 million in 2030, and will continue to 700 million in 2040, representing 10.9% of the global population [1]. As well as, evidence indicates thatobesity may influence human health through excessive energy intake [2]. Overfeeding, as major an unhealthy lifestyle of person, can have a strong impact on diabetes [3]. A study reported that diabetes treatment such as lifestyle changes and hypoglycemic drugs were associated with glucose decreases into the normal range [4], which these studies were conducted in worldwide and found that nutritional therapy were associated with the treatment of diabetic mellitus(DM) effectively [5]. In currently, vegetarian diet, Mediterranean diet and DASH diet have been advocated to play a role in treating diabetes mellitus [6, 7].

There are few studies on dietary patterns on glycemic of patients with type 2 diabetes [8, 9]. Before, a South China research found that fruits and whole grains were associated with lower risk of T2DM [10]. Previous studies targeted the morbidity of T2DM [11], to our knowledge, no such big-sample T2DM patients between dietary patterns and glycemic have been reported based on Chinese community. In addition, the associations between BMI and dietary type with glycemic remain unclear.

At present, this study was based on a large-scale community-based research, of which the participants were enrolled in basic public health service management in Huai'an city, Jiangsu Province in 2018. We conducted a survey based on the dietary habits of 9602 T2DM patients in the past year. We aimed to examine: (1)Explore the dietary status of T2DM population in Chinese community. Dietary pattern is the summary of people's food intake in a period of time, which is a relatively rigorous dietary measurement standard at present. (2) Whether such associations that between diet and glycemic are affected by the regulation of BMI from the T2DM of Chinese. We hypothesized that different dietary patterns would be associated with Fasting Plasma Glucose(FPG), Glycosylated Hemoglobin (HbA1c)control in T2DM, and such association would be have a mediating affections by BMI.

## Methods

### Study object and data source

The project “Comprehensive Research in Prevention and Control of Diabetes Mellitus (CRPCD)” was a community-based, large, ongoing study aiming to exploring an applicable technology for comprehensive intervention in people with T2DM, which was included at baseline investigation of T2DM patients in community chronic disease health management from in Huai'an city, Jiangsu Province. The CRPCD baseline data was collected in December 2013 to January 2014 and demographic characteristics, physical examination (height, weight, waist circumference, blood pressure), daily dietary lifestyle and disease history were self-reported by T2DM patients by investigated under the guidance of professionals. After excluding those who were in poor physical condition and refused to participate in the survey, unqualified questionnaires, some food groups and glycemic deficiency values, a total of 9602 study subjects were enrolled in the data analyses. And extracted fasting venous blood from the patients, what is it used for examining fasting plasma glucose (FPG), high density lipoprotein cholesterol (HDL-C), low density lipoprotein cholesterol (LDL-C), glycosylated hemoglobin (HbA1c), total cholesterol (TC), triglyceride (TG) and etc. This study was reviewed by the ethics Committee of Jiangsu Center for Disease Control and Prevention (No.2013026), and all subjects signed informed consent. The specific measurement methods are shown in the published papers of our research group [12].

### Description of variables

FPG and HbA1c cutoff points according to the Chinese Guidelines for the Prevention and Treatment of Type 2 Diabetes (2013 Edition) [13], abnormal glycemic: FPG ≥ 7.0 mmol/L; HbA1c ≥ 6.5% mmol/L. Daily dietary intake was obtained using a semi-FFQ (food frequency questionnaire) by trained workers, which presents the consumption of each food item among T2DM, who were asked to rate whether they eating or not of rice, grains, fried foods, livestock and poultry aquatic products, egg, dairy products, fresh fruits, vegetable, Bean Products, nuts and cakes. Cumulative smoking of more than 100 cigarettes was defined as smoking, drinking was defined by whether or not they were currently drinking alcohol.

### Statistical analysis

All analyses were carried out using the IBM SPSS Statistics version 20 with the exception of the mediation analyses that used the SPSS PROCESS V3.4, the dietary pattern analysis that performed using Mplus Version 7 [14].

Latent Class Analysis (LCA) was conducted to identify the dietary of113 categories of food that reflect underlying optimal number of dietary types including category probability and latent probability [15, 16], which hypothesizes the existence of dietary patterns with food groups, indicated a general dietary pattern of theT2DM patients.

Demographic characteristics were reported across the latent cluster-3, and Mean ± SD as used to describe the continuous variables with the normal distribution, inter-quartile rangewas used to represent the non-normal distribution data and the frequency (composition ratio) was used to describe the characteristics. Chi-square test for categorical variables and ANOVA for continuous variables were carried out to analyze differences between groups, statistically significance was set at *p* < 0.05.

## Result

### Dietary pattern assessment

We used Latent Category Analysis(LCA) to assigned food groups into the class corresponding to the maximum posterior probability of cluster membership. It can be seen from the Table 1 that AIC, BIC and adjusted BIC continued to decline as the number of categories increased. However, the likelihood-ratio difference test finds that the Cluster—4 is greater than 0.05 and is not meaningful, and thinks that the Cluster—3 model is due to the fourth type. Therefore, considering comprehensively, the 3-cluster is the ideal model. Finally, concluded that when food groups were divided into three modes, the model had the most moderate AIC and BIC, and the BLRT test *P* < 0.001, which was statistically significant, manifesting that our class was successful.

**Table 1:** Fitting indexes of models of different potential categories

Clusters	AIC	BIC	aBIC	Entropy	Loglike- lihood	P for LMR	P for BLRT	Classification	Class probability
2	97,754.097	98,105.414	97,949.699	0.605	-31,320.046	0.000	0.000	4244/5358	0.442/0.558
3	96,245.024	96,775.584	96,540.424	0.633	-48,828.049	0.000	0.000	2714/5479/1409	0.283/0.571/0.147
4	95,979.100	96,688.903	96,374.296	0.672	-48,048.512	0.092	0.000	251/1343/5274/2734	0.026/0.120/0.549/0.285

*AIC* Chachi information criterion, *BIC* Bayesian information criterion, *ABIC* Bayesian information criterion for corrected samples, *LMR* Likelihood ratio test index, *BLRT* Likelihood ratio test based on Bootstrap

**Fig. 1:**
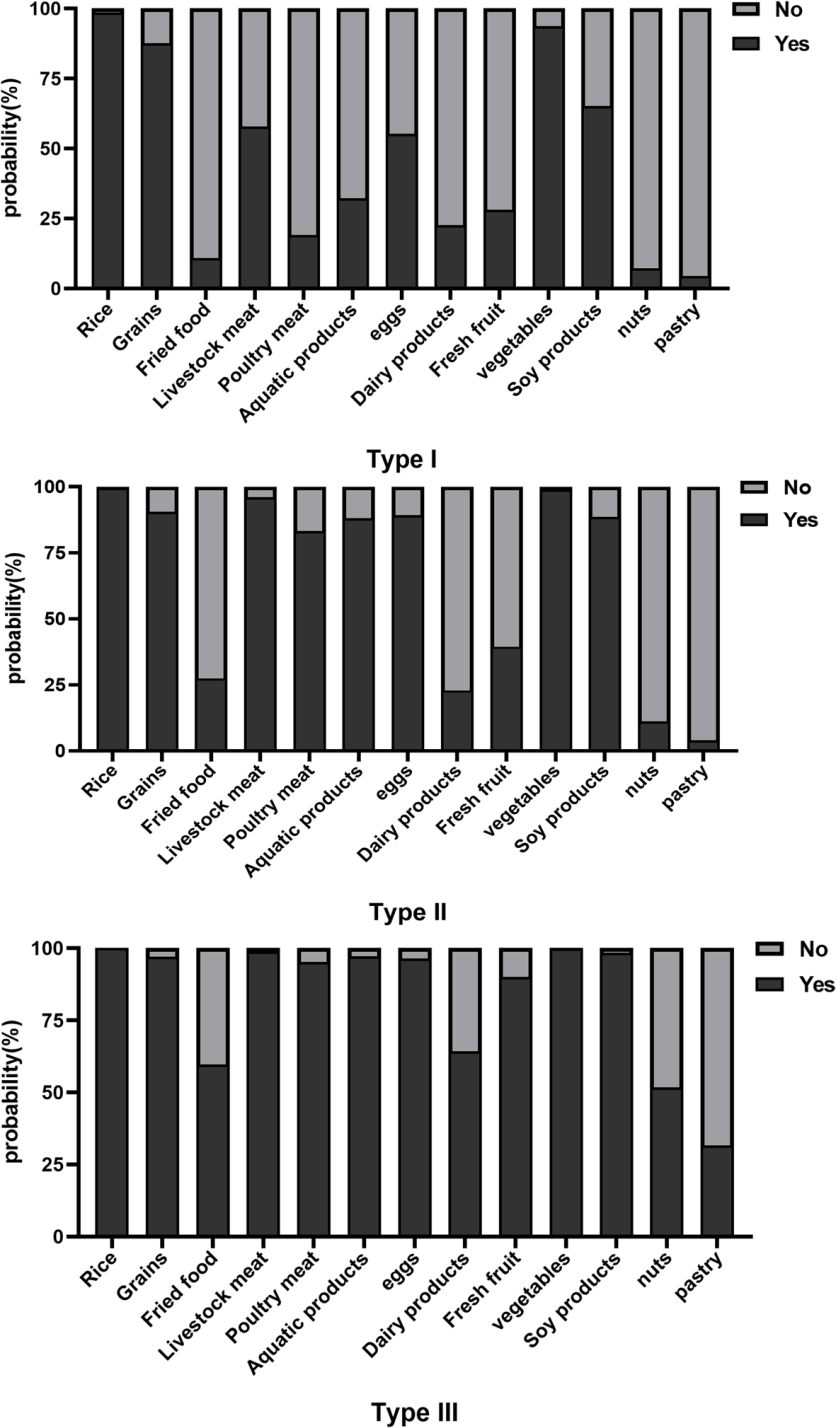
Conditional probabilities of food groups on three potential categories

Based on the best models identified for the 3 potential categories, we obtained conditional probability and category probability of 13 explicit variables related to dietary patterns (Fig. 1). It is not difficult to find that in the three categories, rice, miscellaneous grains, vegetables and so on are the necessities of daily diet intake.The Cluste-3 of scores generated through the LCA were labeled “poor diet”, “moderate diet” and “balanced diet”, and the number of people corresponding to the three dietary types was,1409, 5479 and 2714 respectively. (Table 1).

(1) Type I: it showed the mainly characterized by a high consumption of rice, grains and vegetables and accounting for 14.7%.

(2) Type II: this group accounts for 57.1% and their dietary intake is mainly poultry, livestock, aquatic products, eggs and soy products on the basis of necessities such as rice, cereals and vegetables, and the number of the moderate diet category remained the most through all dietary patterns.

(3) Type III: it is mainly consumed poultry meat, livestock meat, aquatic products, eggs, dairy products, fruits and soy products on the basis of necessities such as rice, cereals and vegetables, which accounted for 28.3%.

### Baseline characteristics

The baseline characteristics of the three potential category groups are shown in Table 2, 9602 type 2 diabetic population including 3623 males and 5979 females. The mean age for study subjects were (61.58 ± 10.09) years, and for the Type I was the highest (63.59 ± 9.69) years. Significant differences were found in gender, age, FPG,HbA1c, educational level, marital status, annual family income, smoking, alcohol consumption, disease course, hypertension and stroke among different dietary patterns (*P* < 0.05).

**Table 2:** Baseline characteristics of different dietary patterns in T2DM

Variable	Dietary type			*χ2*	*P*
	**I**	**II**	**III**		
**Gender**[n(%)]				110.46	< 0.001
Men	355(25.2%)	2183(39.8%)	1085(40.0%)		
Women	1054(74.8%)	3296(60.2%)	1629(60.0%)		
** BMI**(kg/m^2^)	25.69 ± 3.86	25.89 ± 3.60	26.04 ± 3.49	4.34	0.013
** Age**(*x* ± *s*)	63.59 ± 9.69	61.57 ± 9.84	60.56 ± 10.63	11.68	< 0.001
** FPG**(mmol/L)	9.02 ± 4.25	8.98 ± 4.28	8.58 ± 3.76	9.57	< 0.001
** HbA1c**(mmol/L)	7.90 ± 2.10	7.80 ± 2.00	7.70 ± 2.00	7.72	< 0.001
**Literacy**[n(%)]				490.10	< 0.001
No formal education	789(56.4%)	2356(43.3%)	805(29.8%)		
Primary school	391(27.9%)	1616(29.7%)	685(25.4%)		
Junior high school	159(11.4%)	1052(19.3%)	659(24.4%)		
High school and above	61(4.4%)	417(7.7%)	549(20.3%)		
**Marital condition**[n(%)]				50.67	< 0.001
Married	1138(81.5%)	4661(85.7%)	2409(89.4%)		
Unmarried	259(18.5%)	775(14.3%)	285(10.6%)		
**Family income (**[n(%)],Ten thousand Yuan)				224.80	< 0.001
< 10,000 yuan	458(32.7%)	1426(26.2%)	462(17.2%)		
1–30,000 yuan	527(37.6%)	2092(38.4%)	937(34.8%)		
3–100,000 yuan	378(27.0%)	1770(32.5%)	1131(42.0%)		
> 100,000 yuan	39(2.8%)	154(2.8%)	163(6.1%)		
**Drinking**[n(%)]				81.31	< 0.001
Yes	123(8.7%)	980(17.9%)	522(19.2%)		
No	1286(91.3%)	4495(82.1%)	2191(80.8%)		
**Smoking**[n(%)]				21.62	< 0.001
Yes	383(27.2%)	1714(31.3%)	727(26.8%)		
No	1026(72.8%)	3765(68.7%)	1987(73.2%)		
**Course of disease**[n(%)]				30.96	< 0.001
Less than 2 years	424(30.1%)	1704(31.1%)	723(26.6%)		
2–4 years	417(29.6%)	1601(29.2%)	757(27.9%)		
5 to 9 years	298(21.1%)	1241(22.7%)	664(24.5%)		
10 years or more	270(19.2%)	933(17.0%)	570(21.0%)		
**Hypertension**[n(%)]				30.89	< 0.001
No	362(25.7%)	1525(27.9%)	893(33.0%)		
Yes	1044(74.3%)	3938(72.1%)	1817(67.0%)		
**Coronary heart disease** ([n(%)],year)				4.45	0.108
Yes	158(11.2%)	535(9.8%)	303(11.2%)		
No	1165(82.7%)	4663(85.1%)	2286(84.2%)		
Unclear	86(6.1%)	281(5.1%)	125(4.6%)		
**Stroke**[n(%)]				21.64	< 0.001
Yes	236(16.7%)	696(12.7%)	327(12.0%)		
No	1143(81.1%)	4601(84%)	2320(85.5%)		
Unclear	30(2.1%)	182(3.3%)	67(2.5%)		
**Oral hypoglycemic drugs** [n(%)]				37.67	< 0.001
Yes	980(70.4%)	3669(67.3%)	1670(61.8%)		
No	413(29.6%)	1780(32.7%)	1034(38.2%)		
**Insulin therapy**[n(%)]				0.11	0.949
Yes	162(11.6%)	618(11.3%)	306(11.3%)		
No	1231(88.4%)	4831(88.7%)	2398(88.7%)		
** HDL-C**(mmol/L)	1.50 ± 0.50	1.48 ± 0.48	1.45 ± 0.44	6.58	0.001
** LDL-C**(mmol/L)	3.35 ± 1.14	3.34 ± 1.14	3.36 ± 1.08	0.16	0.848
** TC**(mmol/L)	5.42 ± 1.47	5.37 ± 1.43	5.32 ± 1.36	2.84	0.058
** TG**(mmol/L)	2.08 ± 1.81	2.03 ± 1.64	1.93 ± 1.61	4.58	0.010

### Effect of dietary patterns on glycemic control and Glycemic outcome

Different indexes of glycemic control in women with study subjects for different dietary patterns. Participants with the Type I had the highest value of HbA1c and the FPG. Data analysis showed that the control rate of FPG andHbA1c was different among three dietary patterns. There were421 (29.9%), 1798 (32.8%) and 948 (34.9%) FPG controls in the Type I, Type II, Type III, respectively. Moreover,531 (37.7%), 2073 (37.8%) and 1143 (42.1%) had HbA1c control, respectively.

**Fig. 2:**
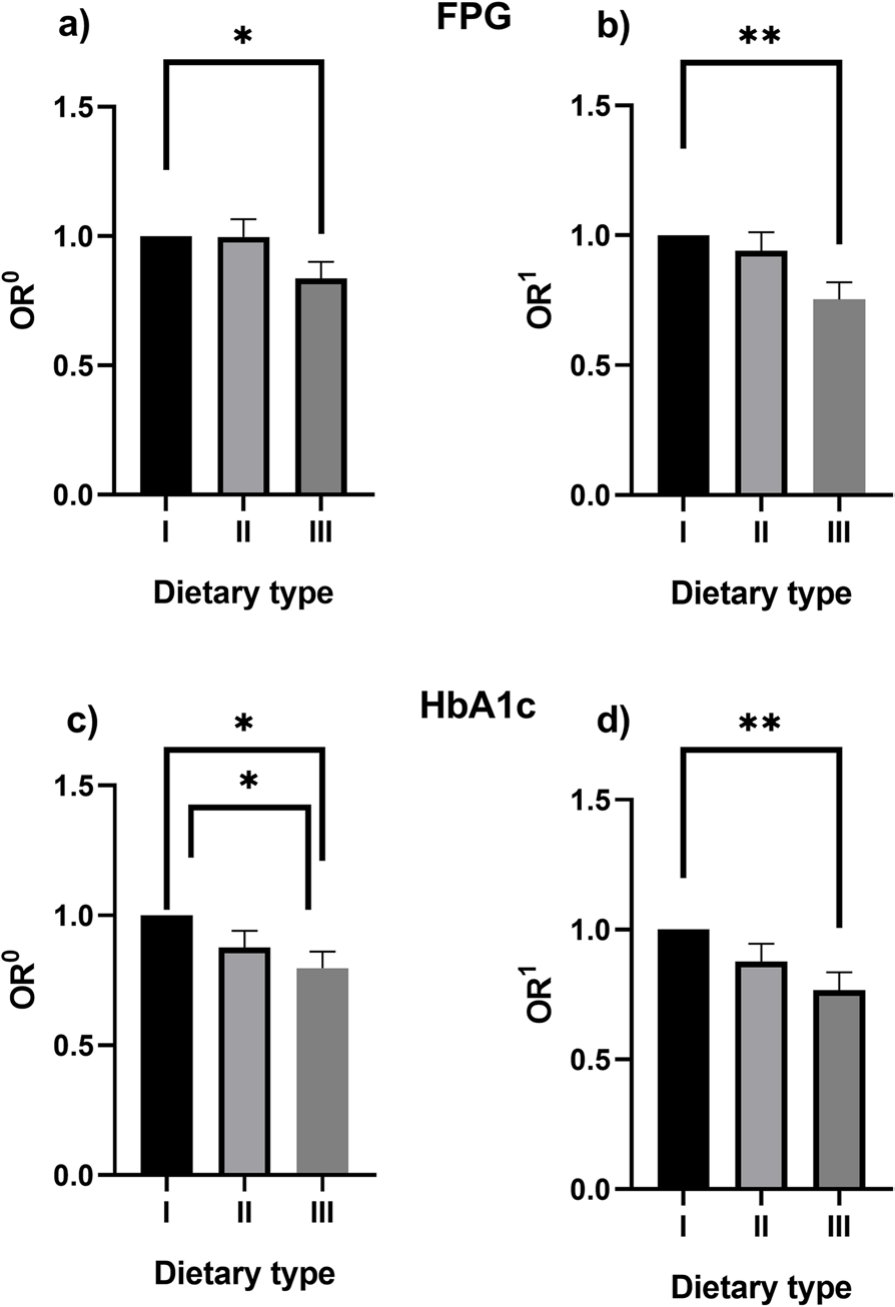
Logistic regression analysis of dietary pattern and FPG and HbA1c. Note:*:*p* < 0.05.VS:Type I.**: *p* < 0.001.VS:Type I.*OR*^*0*^*:* Unadjusted for confounders. *OR*^*1*^: Adjusted for confounding factors such as gender,age,BMI, education level, marital status, family income, smoking, drinking,disease course,HDL-C,LDL-C,TC,TG,oral hypoglycemic drugs, insulin therapy, Hypertension, Coronary heart disease, Stroke

The results of the effect of dietary patterns on different indexes of glycemic control are presented in Table 3 and Fig. 2. After adjusting gender, age, education level, marital status, family income, smoking, drinking, disease course, Hypertension, Coronary heart disease, Stroke. Logistic regression analysis showed that compared to patients with Type I, Type III had a higher control rate of FPG andHbA1c. The OR was 0.75(0.64,0.87),0.76(0.65,0.89), respectively.

**Table 3:** Effects of different dietary types on glycemic control

Indexes of glycemic control	Dietary type	Sample	Controlled	*OR*^*0*^(95%*CI*)	*P*	*OR*^*1*^(95%*CI*)	*P*
	I	1409	531	–	–	–	–
FPG	II	5479	2073	0.99(0.88,1.12)	0.91	0.93(0.82,1.07)	0.31
	III	2714	1143	0.83(0.73,0.95)	0.01	0.75(0.64,0.87)	0.00
	I	1409	421	–	–	–	–
HbA1c	II	5479	1798	0.87(0.77,0.99)	0.04	0.87(0.76,1.00)	0.06
	III	2714	948	0.79(0.69,0.91)	0.00	0.76(0.65,0.89)	0.00

*OR0* Unadjusted for confounders. OR1: Adjusted for confounding factors such as gender,age,BMI, education level, marital status, family income, smoking, drinking,disease

course,HDL-C,LDL-C,TC,TG,oral hypoglycemic drugs, insulin therapy, Hypertension, Coronary heart disease, Stroke

### Mediating effect analysis

**Fig. 3:**
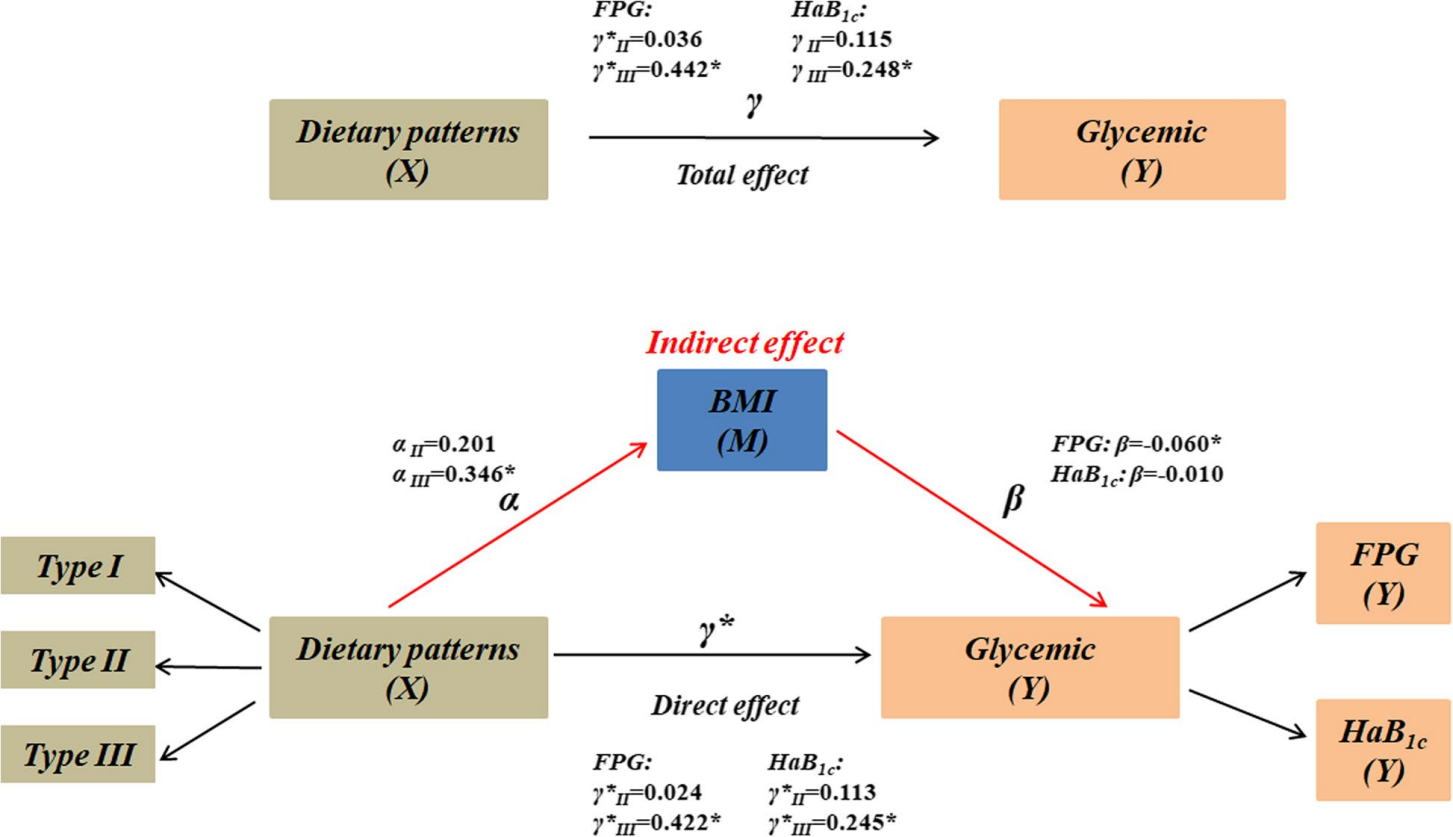
The mediating effect model of BMI on dietary type and FPG and HbA1c

As shown in Table 4 and Fig. 3, when Type I was used as a reference, the mediating effect analysis showed that dietary Type III had a statistically total effect on FPG (γ*_II_ = 0.442, *P* < 0.001). After the addition of BMI, the mediating effect of BMI on FPG in group Type III was -0.021, and the 95% Bootstrap confidence interval was (-0.039,-0.005), which did not include 0, indicating significant mediating effect. The direct effect of Type II on FPG was 0.422, and the 95% Bootstrap confidence interval was (-0.687,-0.156), indicating that the direct effect was also significant. These results indicated that Type III could not only directly affect FPG, but also affect FPG through the mediating effect of BMI. However, the 95% Bootstrap confidence interval of the Type II group was (-0.028,0.002), indicating that the mediating effect was not significant in the group.

**Table 4:** Analysis of the mediating effect of body mass index on diet type and glycemiccontrol

Mediating effect path(compared type I)	Effect	SE	95%*CI*
**FPG**			
TypeII——BMI——FPG	0.012	-0.007	(-0.028,0.002)
TypeII——FPG	-0.024	0.123	(-0.265,0.218)
TypeIII——BMI——FPG	-0.021*	-0.009	(-0.039,-0.005)
TypeIII——FPG	-0.422*	0.135	(-0.687,-0.156)
**HbA1c**			
TypeII——BMI——HbA1c	-0.002	0.001	(-0.003,0.001)
TypeII——HbA1c	-0.113	0.060	(-0.232,0.005)
TypeIII——BMI——HbA1c	-0.003	0.001	(-0.005,0.000)
TypeIII——HbA1c	-0.245*	0.066	(-0.375,-0.114)

*P* < 0.05, which was statistically significant

The total effect between BMI and Type III and HbA1c was -0.248 (-0.378, -0.118), *p* < 0.001, the difference was significant. The direct effect of BMI was 0.245(-0.375,-0.114) and the mediating effect of BMI was -0.003(-0.005,0.000) in TypeIII and HbA1c. The difference has some significance, but the significance is very weak. No mediating effect of BMI was found in TypeII.

## Discussion

We classified T2DM patients into three dietary patterns classes based on their self-reported diet, and indicate. It is found that Type, Type II and Type III of the classes, our results indicate that rice, grains and vegetables were the main food components of each dietary. The trait should be a primary focus in Type I, which we initially thought seemed that related to the economic level, it is more in line with the current low-income population in China, and these rice, grains and vegetables seem to be just the necessary food sources only to maintain the human body. Based on the TypeI, TypeII people will take poultry, livestock, aquatic products, eggs and soy products as the sources of daily dietary intake. Different from the first two groups, in TypeII, people have consumed rice, grains, vegetables, poultry, livestock, aquatic products, eggs and soy products, dairy products, fruits, and so on (Fig. 1).

The Chinese Community research demonstrated that a correlation between different dietary patterns and glycemic, which the balanced diet was a consistent protective factor of FPG and HbA1camong 9602 T2DM patients enrolled in this study,3747 (39.0%) of FPG and 3167 (33.0%) of HbA1c were within the control range. Latent Class Analysis need to be taken into account in the research, in order to accurately explore which diet was more beneficial to control the glycemic in the study subjects. American Diabetes Association with higher adherence to reduce the intake of high-energy and high-fat foods about in 2013 [17]. It is well known that nutritional balance is an important factor in maintaining human health and well-being throughout the lifecycle [18]. Ensuring a balanced diet means having food diversity, which is the reason of food contains a variety of nutrients, and a variety of nutrients in different foods [19].

Our study was able to identify three dietary patterns associated with glycemic control in T2DM patients. Among the three dietary types, TypeIII had a higher glycemic control rate and had a greater reduction in glycemic risk than TypeI. TypeIII significantly reduced FPG (0.75(0.64,0.87)) and HbA1c (0.75(0.65,0.89), which the main feature of dietary intake is greater diversity, suggesting that study subjects should maintain a diversified diet as much as possible in order to better improve the glycemic control rate of T2DM patients.

The relationship between Type III and glycemic may have the following explanations: First, Dietary diversity is considered by nutritionists to be an important factor in improving dietary quality, which can improve the nutritional status of the human body. The richer the diet type, the more balanced the nutrition [20, 21]. Secondly, only when the dietary intake is more abundant, the interaction of various nutrients in the body can maintain balance [22]. For example, dairy products were the main source of calcium and vitamin D, which could help the body better and protect its function [23]. And that fresh fruits are important sources of antioxidants, which can be consumed in moderation to prevent functional damage by reducing inflammation and oxidative stress [24]. Therefore, this suggests that study subjects should be encouraged to intake more and pay attention to their daily diet and eat a variety of foods within the economic range to ensure a balanced diet.

BMI is widely used to measure general obesity and is currently recognized as the best indicator to measure systemic obesity [25]. Our study showed that using TypeI as a control, FPG and HbA1c were associated with TypeIII through BMI, with a mediating effect of -0.021(-0.039,-0.005). A possible explanation is that there may be close relationship between BMI and diet. Besides, there is previous evidence for the associations between each of these factors (dietary-glycemic、dietary-BMI, BMI-glycemic) [26–31]. And in this study, we find that TypeIII can significantly reduce glycemic.

Long-term development of type 2 diabetes is associated with inadequate glycemic control [32]. Previous studies have confirmed that the effects of FPG and HbA1c are different, and study subjects who vegetarian dietary pattern can effectively reduce HbA1c, but has little effect on FPG [33]. Before adjusting confounding factors, TypeII could reduce HbA1c in this study (*P* = 0,04), but had little effect on FPG, compared with TypeI as a control. At the same time, the results of mediation analysis showed that BMI accounted for about 4.98% of the influence between TypeIII and FPG. However, the effect of BMI between TypeIII and HbA1c was small, accounting for about 1.22%, and our mediation analysis found that the mediation effect was also close to the critical value, which may be limited by our sample size and study population.

### Advantages of the research

First, the sample size was large (*n* = 9602 T2DM patients) and the results were reliable, for which dietary data collected over the past year better reflect participants' daily dietary intake status than the three-day weighting method [34]. To our knowledge, this is the first study of analyzing the role of BMI in dietary patterns and glycemic associated with type 2 diabetes in Chinese which has a very novel characteristics.

### Limitations of the research

The current study has several limitations. First, this study is a cross-sectional study, and it is difficult to infer causality. Frequency of self-reported dietary intake by the food frequency questionnaire was prone to recall bias. Second, Analysis of dietary patterns is subject to geographic, cultural, ethnic and economic differences, which is subjective. It is also important to note the limitations of statistical methods. The assignment of food groups to latent classes is based on their highest estimated probability to the identified pattern. Therefore, these underlying patterns should not be considered as actual dietary patterns, but as approximations of more complex patterns. Therefore, prospective studies are needed to further explore the potential mechanism between study subjects diet and glycemic.

## Conclusion

TypeIII contains poultry meat, livestock meat, aquatic products, eggs, dairy products, fruits, soy products, rice, cereals and vegetables, are a relatively diverse diet structure. This study found that dietary diversity can significantly reduce FPG and HbA1c in T2DM patients, which firstly identified and BMI significantly mediated the association between diet and fasting glucose. The findings provided new insights in understanding the possible mechanisms involved in the association between dietary and BMI.

## Data Availability

The data was obtained from Huai 'an Center for Disease Control and Prevention, Jiangsu Province. The data is confidential and cannot be disclosed. If anyone needs data from this study, they should contact corresponding author Enchun Pan.
